# Limited Utility of Serology and Heterophile Test in the Early Diagnosis of Epstein–Barr Virus Mononucleosis in a Child after Renal Transplantation

**DOI:** 10.3390/medicines7040021

**Published:** 2020-04-22

**Authors:** Alexandra Byrne, Rachel Bush, Felicia Johns, Kiran Upadhyay

**Affiliations:** 1 Department of Pediatrics, University of Florida, Gainesville, FL 32610, USA; 2 Division of Pediatric Nephrology, Department of Pediatrics, University of Florida, Gainesville, FL 32610, USA

**Keywords:** Epstein–Barr virus, serology, mononucleosis, renal transplant, pediatric

## Abstract

**Background:** Epstein–Barr virus (EBV) infection is associated with significant morbidity and mortality in renal transplant (RT) recipients. The spectrum of illness ranges from infectious mononucleosis (IM) to post-transplant lymphoproliferative disorder (PTLD). In association with clinical signs and symptoms, virus-specific serology and heterophile antibody tests are widely used in confirming the diagnosis of IM in the general population. However, these tests may have a limited role in immunosuppressed RT recipients from seropositive donor, especially in children who were EBV-seronegative prior to the transplant. The aim of this study is to evaluate the utility of these tests in the early diagnosis of IM in this subset of patients. **Methods:** This is a case study with a review of literature. **Results:** Here, we present a 14-year-old male with hemophilia B who presented with fever, fatigue, sore throat, palatal petechial rash, exudative tonsillitis and cervical lymphadenopathy 3 months post-RT. He was EBV seronegative prior to RT and received a deceased donor kidney transplant from a seropositive donor. Induction was done with Thymoglobulin and maintenance immunosuppression consisted of tacrolimus and mycophenolate. Initial heterophile antibody test (monospot) was negative, but became positive at 5 months and remained positive at 9 months follow-up post-RT. EBV viral capsid antigens (VCA) IgM and IgG, early antigen (EA) and nuclear antigen (EBNA) were all negative at the time of presentation. VCA IgM and IgG both became positive at 5 months and peaked at 9 months follow-up, however the EA and EBNA remained negative. EBV viral load as measured by polymerase chain reaction (PCR) was negative for the first 3 months post-RT but became positive at presentation, peaked at 6 months and started declining thereafter. Peripheral blood smear examination showed no absolute and atypical lymphocytosis. Cytomegalovirus PCR in the blood and throat culture for streptococcus were negative. There was no splenomegaly. He was managed conservatively with intravenous fluids, bed rest, antipyretics and reduction of immunosuppression. **Conclusions:** EBV serological markers have a limited role in the early diagnosis of EBV-IM following RT in prior seronegative children. Initial heterophile antibody test may also be negative, and hence a repeat test may be necessary. Once becoming positive, the VCA IgM may remain persistently elevated for prolonged duration. In addition to the suppressed cellular immunity secondary to immunosuppression, humoral response to viral infections is also delayed in transplant recipients, especially in the early transplant period. Hence, routine monitoring with PCR is superior to serology in diagnosing IM early and monitoring the EBV infection post-RT for timely evaluation and management.

## 1. Introduction

Epstein–Barr virus (EBV), a double-stranded, enveloped DNA virus, belonging to the gamma herpes virus family, is the most common cause of infectious mononucleosis (IM). EBV infects over 90% of the human population worldwide and the rates of infection are higher among ages 15–24 years with 6–8 cases/1000 persons per year [1]. By four years of age, EBV seroprevalence is close to 100% in developing countries and 25–50% in lower socioeconomic groups in the United States [2]. IM is characterized by a triad of fever, tonsillar pharyngitis, and cervical lymphadenopathy [3]. The primary modes of exposure are saliva, organ transplantation or blood transfusion [4,5]. EBV-associated IM is a benign lymphoproliferative disorder in most immunocompetent patients, but may cause significant morbidity and mortality in those who are immunosuppressed, including renal transplant (RT) recipients [6,7]. EBV predominantly infects the epithelial and B cells. In RT recipients, the decreased anti-EBV T-cell immune surveillance, due to usage of immunosuppressive agents, may allow aberrant outgrowth of EBV-transformed B-cells leading to malignant post-transplant lymphoproliferative disorders (PTLD) [8,9,10]. The risk for developing PTLD is especially higher in pre-transplant EBV seronegative recipients, which is true for most young children. The incidence rate of PTLD is 4–22% for the various categories of pediatric organ transplant recipients as opposed to an average of 1–2% in adults [11].

Similar to the non-immunosuppressed patients, the presumptive diagnosis of EBV-associated IM in immunosuppressed RT recipients can be made clinically. However, a definite diagnosis does require demonstration of positive EBV serology and/or heterophile test since IM-like clinical picture can be seen with cytomegalovirus (CMV), human immunodeficiency virus (HIV) and Group A streptococcal infections [12,13], among others. The virus-specific serology poses a challenge, especially in prior seronegative children, due to delayed or absent humoral response [14]. In addition, heterophile tests (monospot) tests are less sensitive early in the course of illness and in young children [15,16]. Hence, relying on these tests only may cause a delay in diagnosis. Quantitative polymerase chain reaction (PCR) to detect EBV DNA in whole blood or serum is used to monitor EBV infection in the RT recipients [17]. Since PCR is independent of the body’s ability to mount cellular or humoral immunity, it can be a more reliable test to confirm EBV mononucleosis in the presence of clinical signs and symptoms. Here, we present a 14-year-old EBV-seronegative adolescent who presented with typical clinical features of IM, negative serology and monospot test, but with positive PCR, three months after RT from a seropositive donor.

## 2. Methodology

Heterophile antibody test was performed by both solid phase immunoassay and by latex agglutination assay. Antibodies to viral capsid antigen (VCA) IgM and IgG, early and nuclear antigens, were performed by semi-quantitative chemiluminescent immunoassay. EBV viral load was measured by quantitative PCR.

## 3. Case Presentation

A 14-year-old Caucasian male with hemophilia B underwent a deceased donor RT for end stage renal disease secondary to ischemic renal injury from intra-abdominal bleeding at birth. Chronic nightly peritoneal dialysis was initiated five months prior to the transplant. Treatment for hemophilia B included factor IX replacement therapy. He did not require blood transfusions due to the neonatal period. He had received all routine immunizations prior to the transplant. Induction was done with a total of 4.5 mg/kg of Thymoglobulin and maintenance immunosuppression consisted of tacrolimus and mycophenolate. He was on a steroid withdrawal protocol. There was an EBV mismatch between the donor and recipient (donor positive and recipient negative for EBV immunoglobulin (Ig) G). His serum EBV VCA IgG was 10.5 IU/mL (not detected: 17.9 U/mL or less) at the time of transplant. Both donor and recipient were CMV IgG positive. He had an immediate recovery of renal function post-RT with baseline serum creatinine of 0.9–1 mg/dL. Monthly viral surveillances for EBV, CMV and BK virus post-RT showed absence of viremia by PCR. Three months post–RT, he presented with fever, sore throat, and fatigue. His immunosuppression regimen at that time included tacrolimus 1.5 mg twice daily and mycophenolate 500 mg twice daily. Serum trough tacrolimus levels were at therapeutic level, as per our institution’s protocol. Anti-infective prophylaxis consisted of sulfamethoxazole-trimethoprim 800–160 mg three times weekly, and valganciclovir 450 mg daily. He had completed a 6-week course of prophylactic nystatin post-RT. Treatment of hemophilia B continued with once weekly factor IX infusions. There were no household sick contacts and he denied oral contact with another person. He had no other post-operative complications or acute rejection and reported adherence to the medications. 

Ten days prior to this presentation, the patient had developed a sore throat and fatigue for a week. Both the rapid streptococcal and heterophile antibody test by latex agglutination were negative at that time. Supportive measures for viral pharyngitis were done; however, over the next 10 days, his symptoms progressed with the development of fever, prompting this presentation. 

**Figure 1:**
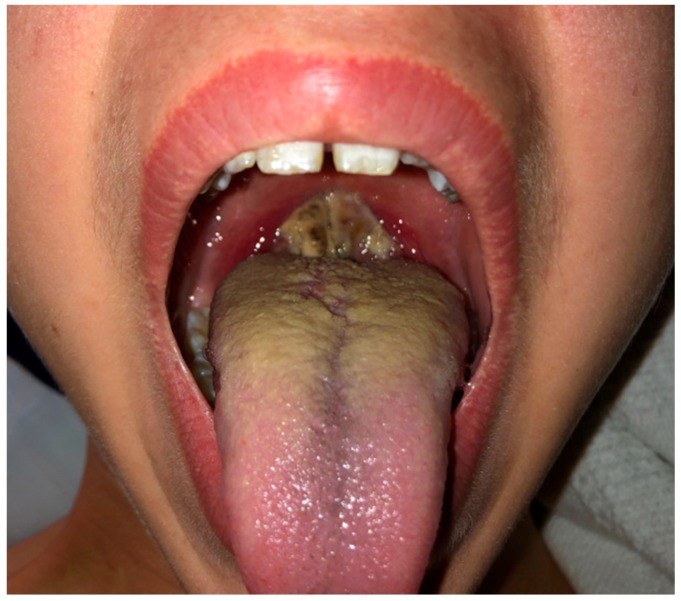
Oropharyngeal examination showing bilateral tonsillar enlargement with exudates.

On examination, his vital signs were as follows: temperature 101.1 ⁰F, pulse 100 beats per minute, respiratory rate 18 per minute, oxygen saturation 99% on room air, and blood pressure 130/80 mm Hg. Height and weight were both at 50th centile. He was ill-appearing and was sitting in a tripod position. Throat and neck examination showed significant bilateral tonsillar enlargement, erythema, and exudate (Figure 1) and tender anterior cervical lymphadenopathy. Abdominal examination showed no tenderness to palpation over his transplanted kidney in his right lower quadrant and an absence of splenomegaly. Investigations revealed elevated serum C-reactive protein of 40 mg/L (0–5 mg/L), white blood cell count of 3.4 × 10^3^/mm^3^, absolute neutrophil and lymphocyte count of 2800 cells/µL and 240 cells/µL, respectively, and atypical lymphocytes of 1%. Hemoglobin, platelet count and liver function test were all in normal range. Renal function test showed serum creatinine of 1.2 mg/dL (baseline 0.9–1 mg/dL) with stable electrolytes. Given EBV discordance (donor EBV + and recipient EBV −) along with fever, exudative tonsillitis, cervical lymphadenopathy and fever, there was a high index of suspicion for IM secondary to EBV. However, the heterophile antibody test by both solid phase immunoassay and latex agglutination assays was negative again. Furthermore, the serologic tests were all negative (EBV VCA IgM < 10 U/mL (not detected: 0–43.9 IU/mL), VCA IgG 15 IU/mL (not detected: 0–17.9 IU/mL), early antigen (EA) IgG < 5 IU/mL (not detected: 0–8.9 IU/mL), nuclear antigen (EBNA) IgG < 3 IU/mL (not detected: 0–17.9 IU/mL)). Serum CMV PCR and BK virus PCRs were negative. Whole blood EBV quantitative PCR showed EBV viral load of 1970 copies/mL. (undetectable: <390 copies/mL; ARUP laboratories, Salt lake City, UT, USA). Prior EBV PCR loads were undetectable at one and two month post-RT follow-up appointments. *Bartonella henselae*, *Bartonella quintana, Toxoplasma gondii* IgG and IgM antibodies along with serum adenovirus PCR were all negative. Serum tacrolimus trough level was at goal (6–7 ng/mL, as per our institution’s protocol). Hepatitis screen, HIV, antinuclear antibody, and rheumatoid factor were negative. Peripheral blood smear did not show evidence of blast cells. Abdominal sonogram showed normal appearing renal transplant with no splenomegaly. Throat culture was negative for beta-hemolytic streptococcus, *corynebacterium diphtheriae*, fungus, chlamydia and gonorrhea. Nasopharyngeal swab test for influenza and other respiratory viruses were negative. Due to tripod position on examination, X-ray along with computed tomography scan of the neck without contrast were done which showed narrowing of the airway due to soft tissue swelling but without any peritonsillar or retropharyngeal abscess. However, his respiratory status remained stable on room air throughout without the need for supplemental oxygen therapy. Steroid therapy was not required. He also developed acute hemoptysis, most likely secondary to tonsillar friability in the setting of low factor IX levels, prompting administration of an additional dose of factor IX with resolution of hemoptysis. He was treated with acetaminophen, intravenous fluid therapy and 3rd generation cephalosporin, the latter was discontinued after the throat and blood bacterial cultures resulted negative. Mycophenolate dose was decreased to 250 mg twice daily. He was discharged to home on sixth day of admission once he became afebrile and adequate oral intake was ensured. Discharge medications consisted of tacrolimus, mycophenolate, valganciclovir, TMP-SMX and factor IX replacement therapy. 

**Figure 2:**
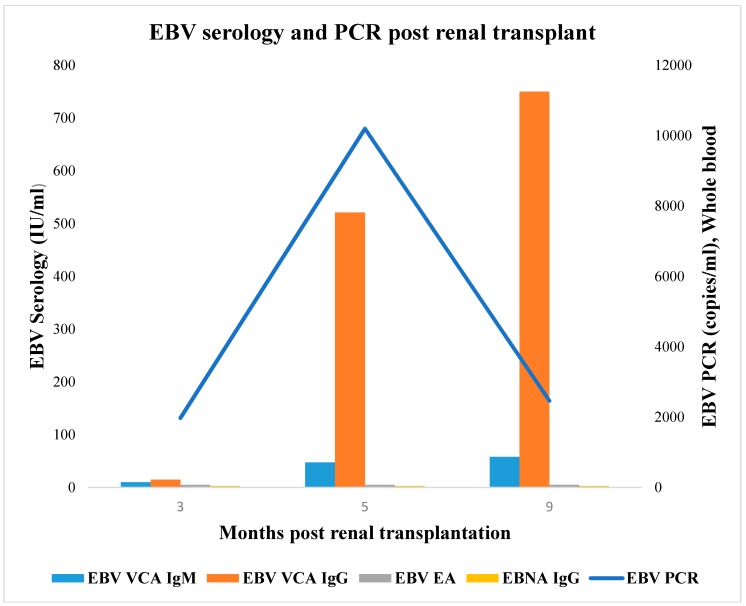
Timeline of Epstein–Barr virus (EBV) serology and polymerase chain reaction (PCR) post-renal transplantation. EBV serologies (viral capsid antigen (VCA) IgM, VCA IgG, early antigen (EA) and nuclear antigen (EBNA) IgG expressed in IU/mL) and EBV DNA PCR expressed in copies/mL.

At subsequent outpatient clinic follow-up visits, whole blood EBV PCR showed a persistent rise to a peak level of 10,200 copies/mL at 6 months, and then gradual decline to 2460 copies/mL at 9 months post-RT (Figure 2). VCA IgM and IgG both became positive at 5 months and subsequently peaked at his most recent 9-month follow-up (Figure 2). Monospot test (latex agglutination) also became positive at 5 months and remained positive at the 9 month post-RT follow-up. Clinical examination showed persistent mild sore throat, tonsillar enlargement and cervical lymphadenopathy until about 5 months when the symptoms started resolving. At his most recent 9-month follow-up visit, he was asymptomatic with resolution of tonsillar enlargement and cervical lymphadenopathy. Renal allograft function remained stable. Serum CMV and BK virus PCRs remained negative. 

## 4. Discussion

EBV infection in transplant recipients may be a primary infection or a reactivation of a prior latent infection [8,18]. Primary infection occurs mainly via exchange of saliva or sharing household items from close-contacts, transplantation of kidney from a seropositive donor in a seronegative recipient or through blood transfusions [19,20]. Reactivation occurs in those with latent EBV and then undergo active viral multiplication in the setting of immunosuppression post–RT. The serologic diagnosis of reactivation/reinfection requires a 10-fold increase in anti-VCA IgG or the presence of anti-VCA IgM in previously IgG seropositive patients [21]. Our patient did not have a history of saliva exchange, sick contacts, and blood transfusions; hence he most likely had a primary infection through the transplantation from the seropositive donor. A single center study of pediatric solid organ transplant (SOT) recipients showed that the seroconversion rate in prior seronegative pediatric liver transplant patients was >75% while reactivation occurred in 50% of prior seropositive recipients [22]. Patients with primary infection had more viral shedding than those who were seropositive at the time of transplant, and heart transplant recipients had higher levels of peak shedding than renal allograft recipients [23]. In terms of risk factor for development of PTLD, primary EBV infection has been shown to be much more of a risk than reactivated infection [24]. Ho et al. described 11 children with EBV-associated lymphoproliferative syndrome post-SOT and 10 had a primary infection. These children were at greater risk due to them being seronegative for EBV prior to the transplant who then acquired primary infection with the transplant from seropositive donor [22]. Other studies have similarly shown that recipients who are seronegative at the time of transplant are more likely to be symptomatic and have EBV related complications, such as PTLD [6,7,25]. 

The most commonly used tests to diagnose EBV-associated IM include heterophile test, serology and PCR. Paul–Bunnell test, the original heterophile antibody test, utilized sheep red blood cells (RBC) while most of the currently used heterophile antibody or the Monospot tests use horse RBCs with improvement in the test if these RBCs were first absorbed to guinea pig kidney or bovine RBCs before sera were added [26]. In immunocompetent patients, initially, the B cells produce polyclonal antibodies in response to EBV infection which are directed against viral and unrelated antigens found on sheep and horse RBCs. The antibodies against the latter antigens, termed heterophile antibodies, comprise only about 5% of total polyclonal antibodies [27]. They are mostly IgM antibodies that do not cross-react with EBV antigens and are the basis of heterophile antibody test [28,29]. Later in the course of EBV infection, these B cells are kept in check by the recruitment of cytotoxic CD8+ T cells which provide a polyclonal regulatory response, and are an important part of host defense mechanism against EBV [30]. Since the peak levels of virus-specific IgM and IgG response usually occurs only around 6–17 days after the onset of clinical symptoms, the serology may not be helpful in the early stage of presentation [31]. The incubation period of IM is usually 2–7 weeks and since the heterophile antibodies are seen 2–9 weeks after the person is infected, heterophile antibody tests may be preferable for early diagnosis at the time of presentation. Commercial test kits for these antibodies (by solid phase immunoassay or latex agglutination assay) are available and have sensitivities of 70–92% and a specificity of 96–100%, with a lower sensitivity in the first two weeks of illness [32]. The false negativity rate of these tests could be as high as 25% in the first week, 5–10% in the second week, and 5% in the third week [15]. Latex-based heterophile antibody assays may be a little more sensitive than the solid phase assays but the specificity is similar between the two [33]. Our patient was tested with both solid phase and latex agglutination assays. In addition, in younger patients less than 12 years of age, the test sensitivity is only 25–50%, as compared to 71–91% in older patients [15]. Horwitz et al. showed that children 1–2 years of age were significantly less likely to have positive heterophile antibody tests compared with children 2–4 years of age (27.3% vs. 76.2%) [16]. Therefore, heterophile tests may only be appropriate for use in older children. Once formed, these antibodies disappear in few weeks but in some, they may persist for up to a year after infection [28]. Hence, heterophile antibody tests may help diagnose IM retrospectively in some patients, if the initial test was negative, as in our case. Moreover, the antibodies detected by the test can be caused by conditions other than IM (for example, CMV, hepatitis, rubella, HIV, autoimmune conditions, adenovirus) due to cross reactivity with these conditions [32]. Our patient had negative CMV and adenovirus PCRs along with negative hepatitis and HIV tests. Due to these limitations, Center for Disease Control and Prevention in Unites States does not recommend the heterophile test for general use in the diagnosis of IM, instead suggesting viral serology as a method of choice [34]. 

The initial serologic response to EBV infection occurs with the production of IgM antibody to the VCA in about 75% of patients [35], which usually disappears within 4–6 weeks. In immunosuppressed patients, however, the viral shedding may be prolonged, symptoms may last longer and be more severe [36]. Hence, once the intense immunosuppression period of T lymphocyte depletion is over (usually few months post-transplant), in the setting of persistent viral shedding, the newly produced host T cells will continue to stimulate B cells to produce virus-specific IgM and IgG. This could have led to the delayed onset but more prolonged presence of IgM antibody in our patient. Anti-VCA IgG also appears in the acute phase of EBV infection, peaks at 2–4 weeks after onset in most patients, declines slightly and then persists for the rest of a person’s life. Hence, the presence of VCA IgG indicates that the infection has occurred in the past. Antibody to early antigen (EA) is present in 60–80% only, and may indicate active viral replication and is especially helpful if the heterophile antibody is negative and VCA IgG or IgM yields inconclusive results. Of note, 20% of healthy individuals can have EBV EA antigen [35]. Anti-EA IgG generally falls to undetectable levels after 3–6 months. Antibody to nuclear antigen (EBNA) is not seen in the acute phase but slowly appears 2–4 months after the onset of symptoms and persists for the rest of a person’s life [37]. The infection then remains latent. Hence, the comparison of paired sera from acute and convalescent period showing presence of EBNA IgG in the absence of VCA IgM rules out the acute infection. With regards to the efficacy of serology, Bruu et al. analyzed six commercial tests for EBV-specific antibodies, with sensitivities ranging from 95–100% and specificities ranging from 86–100% [38]. Hence, EBV-specific serology appears to be more sensitive but less specific than heterophile antibody testing. The reduced specificity is due to significant cross-reactivity of VCA-IgM to other infections such as CMV [38]. On the other hand, increased sensitivity of serology, including IgM, can produce false positive results [39].

Similar to the delayed serological response after immunization in SOT recipients [40], serology unfortunately is unreliable as a diagnostic test for either PTLD or primary EBV infection in transplant recipients [8]. These patients show a marked delay in their humoral response to EBV antigens, and many fail to develop IgM antibodies altogether [8,14,41]. Both T and B cells are required to induce an antibody response. T follicular helper cells provide seven main forms of T cell help to B cells: signals that promote survival, proliferation, plasma cell differentiation, hypermutation, class-switch recombination, adhesion and chemo-attraction (cell migration) [42]. Hence, in the absence of cytotoxic T cells secondary to usage of lymphocyte-depleting immunosuppressive agents, the humoral response is also inhibited along with cellular immunity [42]. This state of T cell anergy allows EBV to escape the immune elimination by utilizing a distinct latency expression programs to establish a persistent infection and potentially leading to PTLD [30,43]. It is important to remember that T cell anergy can also occur due to T cell receptor-specific impairment in the induction of genes involved in T cell proliferation, allowing uninhibited proliferation of infected B cells, in some immunocompetent patients with acute IM [44]. Regardless of the mechanism of anergy, the antibody response is reduced and in some cases may be selective. Diminished or absent antibody response to EBNA may be observed despite persistence of anti VCA IgG. Cen et al. described a markedly reduced antibody response to the latent cycle antigens, EBNA 1, EBNA 2 and EBNA-LP in immunosuppressed SOT recipients with PTLD, but not to the lytic cycle VCA when compared to the normal immunocompetent controls [45]. At 9-month follow-up, our patient had not been able to produce anti-EBNA IgG although the VCA IgG had reached its peak level. Hence, the typical pattern of appearance of serological markers may not be evident in these patients. 

EBV PCR measures the quantitative viral load in the blood and is the most sensitive test for detecting EBV infection in the post-transplant period [17]. A variety of blood specimens have been used for EBV load quantification, including unfractionated whole blood, peripheral blood mononuclear cells, and plasma. However, the whole blood EBV DNA is believed to be superior to plasma EBV DNA as the cell-associated EBV can be detected well before the plasma DNA is detected. Additionally, the cell DNA may be present at high levels without even being present in the plasma [21,46]. The whole blood sample is also simple to process and provides quantification of both cell-associated and cell-free virus, yielding an absolute viral load, irrespective of concerns about fluctuations in cell number, lysis and loss during separation [17]. Hence, transplant clinical guidelines suggest monitoring of high-risk patients for EBV-related PTLD by nucleic acid testing post-RT instead of serology [47]. In a large, retrospective study of the incidence of PTLD in RT recipients in the United States, the risk of PTLD was more than six times higher for donor+/recipient- deceased donor transplants compared with R+ transplants [48]. However, there are no clinical guidelines that exist for diagnosis of EBV-IM without PTLD in the post-RT period. Since the EBV serology and heterophile tests may be negative in the early post-RT period, this report emphasizes the value of repeat testing of serology and heterophile tests to establish a diagnosis of IM. It is important to know that since low level detection of EBV by PCR is common in transplant recipients [49], the mere presence of a positive viral load does not always indicate mononucleosis in the absence of the classic presentation such as lymphadenopathy, pharyngitis and fever. Hence, the serial testing of serology and heterophile tests may have a role in retrospectively establishing a diagnosis of IM in these patients. 

Other tests that may be useful in diagnosis of IM are lymphocyte to white cell count ratio, real-time PCR targeting various EBV genes, an absolute increase in the number of peripheral lymphocytes, and atypical lymphocytes [50,51,52]. Studies on lymphocyte to white cell count ratio as a diagnostic marker of IM has shown conflicting results [50]. Usage of raised absolute lymphocyte count has been suggested as providing a quick clue to the diagnosis of IM while awaiting for serology or heterophile antibody test result [52]. The atypical lymphocytes are primary T cells, produced in response to the EBV-infected B cells [29]. Commonly, absolute lymphocytosis (>50% lymphocytes) in the presence of >10% atypical lymphocytes are considered diagnostic of IM. The case presented in this report did not have absolute lymphocytosis with only 1% atypical lymphocytes, most likely secondary to lymphopenia from immunosuppression. Hence, lymphocyte-based assays are unlikely to be useful in transplant recipients, especially in the early post-transplant period. 

## 5. Conclusions

The EBV serology may underestimate the active presence of EBV in seronegative SOT recipients. Furthermore, in the absence of positive heterophile antibodies, the confirmatory diagnosis of mononucleosis becomes much more difficult. Not only has the EBV DNA load in the blood allowed risk assessment of development of EBV-associated disease in immunosuppressed patients, it also is helpful in making a definite diagnosis of IM in the presence of clinical signs and symptoms.
